# Ophthalmology

**Published:** 1900-05

**Authors:** Alvin A. Hubbell

**Affiliations:** Buffalo, N. Y.


### OPHTHALMOLOGY.

Conducted by ALVIN A. HUBBELL, M. D., Buffalo, N. Y.

### OPERATIVE TREATMENT OF SMALL ERRORS IN THE CURVATURE OF THE CORNEA.

REYMOND, of Turin, in discussing this subject (*Klinische Monatsblätter für Augenheilkunde*, November, 1899,) says that various attempts, more or less successful, have been made to reduce the amount of astigmatism in an eye by judiciously placed incisions in the corneo-scleral margin, and even by cautious removal of minute portions of the corneal substance; thus Baiardi, by repeated small incisions at both ends of the meridian he wishes to affect, claims to be able to reduce an astigmatism as much as 1.5D. By means of operations on the rabbit he found that the amount of effect was proportional to the size of the incision and its nearness to the center. Lucciola, of Turin, has attempted in a number of cases to reduce the amount of astigmatism present after cataract extraction, by means of incisions running very obliquely through the extreme periphery; these appear to have the effect rather of increasing the curvature in the affected meridian during the next day—a result, it will be noticed, which is exactly contrary to that of incision through clear cornea. Lans has made a series of very interesting investigations regarding the effects produced by nonperforating wounds of the cornea in rabbits; he observes an increased curvature in the meridian parallel to the wound, and in a certain number of instances a flattening in that at right angles to it. During and after cicatrisation the astigmatism diminished considerably, but he believes that by making two "parallel" incisions with a cautery, each involving about two-thirds of the thickness of the cornea and about 2 mm. from the limbus cornea, one at either extremity of a given meridian, he can produce a permanent astigmatism of from 4 to 6 D. In a certain number of cases of conical cornea Reymond has been able, by simple incision, to cause complete disappearance of the central opacity, which owes its origin to edema of the tissue, and at the same time to check the process of ectasia; he has been much pleased, also, with the results in this disease of making, at the base of the central portion of most acute curvature, a puncture and counter-puncture with a Graefe knife, which is then withdrawn without completion of the incision, the process being repeated two or three days later in the opposite direction. This seems to point to the possibility of causing some flattening down, or lengthening of the radius of curvature by incisions near the center; but at the close of his paper Reymond admits that the results of all such interference are so uncertain in the case of ordinary astigmatism, and in order that an operation may be even reasonably effective, one must attack the cornea so near the center, and thus leave an unsightly scar, that he does not see his way to recommend any operative treatment of a merely optical error.

With this conclusion most practical surgeons will be inclined to agree.

### CORNEAL HORN.

Mr. Arnold Lawson (*Ophthalmic Review*, March, 1900,) read a paper at the meeting of the Ophthalmological Society of the United Kingdom, in January, 1900, founded on a case of corneal horn, which he claimed to be unique as far as the literature of the subject was concerned. The case occurred in a female hydrocephalic idiot, aged eight, the horn being situated on the middle of the cornea and about half an inch long. The eye being unsightly as well as totally blind, he had excised it, and now displayed sections of the growth. He considered that it was a true cicatricial horn, composed of granulation tissue, and he thought the sequence of events was as follows: (1) Atropic changes following pressure on the ophthalmic division of the fifth nerve; (2) ulceration of the cornea, leading to perforation; (3) the formation of granulation tissue, undergoing unhealthy proliferation, similar to that which produced "proud flesh." He maintains that the tumor consisted not of heaped-up corneal epithelium but of granulation tissue, which, in absence of an epithelial covering, went on proliferating without a check.

Mr. Treacher Collins suggested a different explanation—namely, that after sloughing of the cornea, a mass of granulation tissue formed on the surface of the iris, which developed into a pseudo-cornea and became staphylomatous. The unusual thing was the accumulation of epithelium on the surface of this which gave rise to the horn-like appearance.

---

### USE OF ATROPIN SULPHATE AS A MEANS OF DIAGNOSTICATING HEADACHES CAUSED BY EYE-STRAIN.

Dr. Landman, of Toledo, (*The Journal of the American Medical Association*, April 14, 1900,) believes that headaches which are most frequently produced by ocular defects are situated, as to their frequency, in the following regions: supraciliary, occipital, occipito-frontal, vertical and temporal.

When there is a general headache, that is, one equal in all parts of the head, it is due to some depraved state of the system. He alludes to one kind of a headache—namely, one situated on the top of the head, the vertex, having an area about the size of a silver dollar, circumscribed, and associated with tenderness of the scalp, which is almost invariably due to eye-strain, and often amenable to treatment by the use of glasses. The *clavus hystericus* belongs to this class.

Dizziness, when caused by eye-strain, will often be relieved by paralysis of the ciliary muscle with atropin sulphate, and can be permanently abolished by glasses. The conclusion is that when a persistent headache, situated in any of these localities, disappears on atropin being used in the manner directed, the cause is probably due to eye-strain and can be remedied by properly adjusted glasses. To obtain the result, the atropin must be pushed to the complete suspension of accommodation. The directions for its use are simple: A solution of one fourth of a grain of sulphate of atropin to two drachms of water, with directions to drop three drops in each eye, three times a day, until ten instillations have been made. When, on the return of the patient after the use of the atropin, we find that the headaches have disappeared, then a favorable prognosis for the dissipation of the headaches, through correcting lenses, can be given. There is one general caution to be observed, and that is the careful use of atropin in older persons. In increased ocular tension it should never be used.

---

STREPTOSYPHILIS.

M. BOUCHERON (*Ophthalmic Review*, March, 1900,) read a paper at a meeting of the French Society of Ophthalmology, October, 1899, in which he held that microbic associations in syphilitic lesions are now recognised to exist, and among these streptosyphilis is one of the most interesting. In some instances the streptococcal lesion has existed previously to the syphilitic infection, and one then sees a lesion develop which is the compound result of the double general or local infection. This variety of mixed lesion is of particular moment in regard to affections of the eye, etc., and it is necessary under such circumstances to treat both morbid conditions coincidently. He carried this out in a certain patient, thus: A man had been under his care for dacryocystitis which had resisted other treatment but yielded to injections of antistreptococcic serum. Some little time after this he acquired syphilis, followed early by violent and very painful iritis. This was treated with the utmost success without confinement even to the house, by the addition of antisyphilitic treatment to the antistreptococcic. He related several other cases, similar in regard to the undoubted presence of both types of dyscrasia, in which the mixed treatment effected results which could not, in his opinion, have been otherwise attained. The serum was given in doses of one-fourth or one-half c.cm., daily, injected under the skin of either the abdomen or arm. Redness, swelling, etc., occurred locally, if not immediately, then, after the third or fourth injection; later, the local reaction was less, and larger doses could be tolerated. He was of the opinion that the serum possessed a strong antagonistic power against streptococci and their toxine, but was also of virtue as a tonic and stimulant of the nervous system.

